# The Book World of Medicine and Science

**Published:** 1903-11-21

**Authors:** 


### THE BOOK WORLD OF MEDICINE AND SCIENCE.

MANUAL OF INTRAGASTRIC TECHNIQUE. By G. HERSCHELL, M.D. Lond. (London: H. J. Glaisher. 1903. Pp. 166 With illustrations. Price 7s. 6d. net.)

THE rapid strides gained in recent years in our knowledge of the diseases of the stomach, have been largely due to improved methods in intra-gastric technique, and any new work on this important branch in the practice of medicine claims for itself the attention of the profession. The author of the present work begins with a description of the stomach tube, and unhesitatingly recommends the pattern of Van Valsah and Nisbet. He proceeds to give an account, in detail, of its useful application for diagnostic purposes, in inflation of the stomach and in extraction of the stomach contents, as well as of its therapeutic use in lavage. The instructions given in passing the tube should prove of great service. Some simple test-meals and practical methods of investigating the nature of the stomach contents, which are described together with the inferences to be drawn, are also likely to be helpful. It will, no doubt, come as somewhat of a shock to some clinicians to read on p. 57 that "the tests for free hydrochloric acid to which we have hitherto pinned our faith, viz., phloroglucin-vanillin and resorcin-sugar, are absolutely fallacious and useless for clinical purposes. It has been pointed out by Faulkner that the necessary heating will produce hydrochloric acid in sufficient quantity to develop the colour in most stomach contents which were HCl. free at the commencement. For instance, any lactic acid present will decompose sodium chloride with the evolution of HCl. Oxalic acid, bin-oxolate of potash, and tartaric acid will do the same." However, Dr. Herschell describes some tests which are both simple and reliable, for the details of which readers must be referred to his book. The chapter which is devoted to lavage of the stomach is replete with valuable hints and instruction. Although Dr. Herschell's methods are described with such minute care as to be of the greatest practical value, yet it must be admitted that in some respects he keeps closely to the ideal in everything, and we think that it would hardly repay anyone but a "stomach specialist" to invest in all the apparatus that Dr. Herschell recommends. However, it is always a good thing to know what is the very best way of carrying out any particular method of investigation or treatment, even if practical considerations force us to be content with less perfect technique. There is no doubt that a perusal of the book will well repay every student or practitioner who desires instruction in methods of examination of the stomach or of treating its various affections.

THE MEDICAL EXAMINATION FOR LIFE ASSURANCE: WITH REMARKS ON THE SELECTION OF AN OFFICE. By F. DE HAVILLAND HALL, M.D., F.R.C.P. Third edition, greatly enlarged. (Bristol: John Wright & Co. 1903. Pp. 112)

THIS third edition of a well-arranged and replete little book has been added to considerably. The points that have to be kept in view in the acceptance or rejection of a candidate for life assurance, or in fixing the rate of premium, are very many, and are affected by changes in medical doctrine. Deaths from phthisis are said to be the chief disturbers of actuarial calculations; so that here the medical adviser has to walk warily. Dr. de Havilland Hall is of opinion that the recent tendency is to make more of the risk of contagion and less of the risk of heredity; but the inquiries under the latter head continue to be of the most searching kind. The probability of cancer is another difficult question, upon which the author writes judiciously, following a paper on the subject by Mr. Thorburn. An "exceedingly difficult" question is that of Habits, all the more difficult that the data are not always accurate. It is suggested that the tobacco habit should become a subject of inquiry, in view of the great increase of cigarette smoking. The important factor of foreign climate is disposed of briefly by reference to certain conventional zones coloured on a map of the world. But in that matter, as well as in the rest, this little book does not profess to be a treatise, but only a concise review of the several things which the medical referee must keep before him so as to deal with them out of his own knowledge. That object it appears to us to fulfil very satisfactorily.

LECTURES ON MASSAGE AND ELECTRICITY IN THE TREATMENT OF DISEASE. By THOMAS STRETCH DOWSE, M.D.Aberd., F R C.P Edin. Fourth and revised edition. (Bristol: John Wright and Co. 1903. Pp. xii., 454. With 101 diagrams in the text. Price 7s. 6d. net)

THIS fourth edition calls for no critical comment. The author is "still of opinion" that massage is an important aid in treatment, and he is gratified that the profession in this country is gradually, but surely, giving greater attention to it, especially in chronic diseases. The descriptions of its applications to various regions of the body, and in various diseased states, are interspersed with much physiology and anatomy from reputable sources for the elucidation of its theory. There is a chapter on the Nauheim treatment of cardiac disease. The last hundred pages are occupied with the subject of medical electricity, and illustrated by numerous figures of apparatus. There is an index of ten pages, as well as summaries of the contents of chapters.

GOLDEN RULES FOR DISEASES OF CHILDREN. By GEORGE CARPENTER, M.D. Lond., M.R.C.P. (Bristol: John Wright and Co. 1903. Pp 167. Price 2s.)

THE name of the author of this little handbook is a sufficient guarantee of the excellence of the work, and the appearance of a second edition so soon after the first is evidence that the book is appreciated. A great deal of very useful information is condensed into its pages, and is presented in such a way as to be readily accessible for reference. We do not hesitate to say that the book will prove most useful both to medical practitioners and students.

